# A Comparison of Two Motion Sensors for the Assessment of Free-Living Physical Activity of Adolescents

**DOI:** 10.3390/ijerph7041558

**Published:** 2010-04-06

**Authors:** Roman Cuberek, Walid El Ansari, Karel Frömel, Krzysztof Skalik, Erik Sigmund

**Affiliations:** 1 Center for Kinantropology Research, Faculty of Physical Culture, Palacky University in Olomouc, Czech Republic; E-Mails: karel.fromel@upol.cz (K.F.); erik.sigmund@upol.cz (E.S.); 2 Faculty of Sport, Health and Social Care, University of Gloucestershire, Gloucester GL2 9HW, UK; E-Mail: walidansari@glos.ac.uk; 3 Academy of Physical Education, Katowice, Poland; E-Mail: k.skalik@awf.katowice.pl

**Keywords:** pedometer, accelerometer, validity, monitoring, measurement, step counter, categorization of physical activity

## Abstract

This study assessed and compared the daily step counts recorded by two different motion sensors in order to estimate the free-living physical activity of 135 adolescent girls. Each girl concurrently wore a Yamax pedometer and an ActiGraph accelerometer (criterion measure) every day for seven consecutive days. The convergent validity of the pedometer can be considered intermediate when used to measure the step counts in free-living physical activity; but should be considered with caution when used to classify participants’ step counts into corresponding physical activity categories because of a likelihood of ‘erroneous’ classification in comparison with the accelerometer.

## 1. Introduction

In order to assess the level of physical activity (PA) and evaluate PA interventions [1], measures are required that have high validity and reliability, whilst being practical and nonreactive [2]. Validity is considered the most important attribute, but for PA assessment tools, and particularly for free-living PA, verifying validity is difficult task [3].

PA is the sum of all those partial bodily movements realized by a muscle-skeletal system and resulting in energy expenditure [4]. Using this definition, appropriate measures are required to assess the levels of attainment of such bodily movements. For this purpose, the techniques used in a practice comprise three groups [2]: criterion standards (direct observation, doubly-labeled water, and indirect calorimetry); objective techniques (heart rate, motion sensors: pedometers and accelerometers); and subjective techniques (self-reported questionnaires, interviewer-administered questionnaires, proxy-reporting questionnaires and diaries).

Although the optimal technique of accurate assessment of free-living PA of children and adults is difficult to ascertain, direct observation is still the most acceptable [2]. However, the use of direct observation to assess free-living PA is hindered by that the technique entails considerable time and personnel requirements [5–7]. Hence, questionnaires and/or motion sensors (MS, e.g., accelerometers and pedometers) are frequently employed instead. Such measurement tools have had their validity [2,8–11] and reliability [2,12,13] verified under various conditions. For instance, De Vries and colleagues [14] provided summaries of methodological studies published between 2004 and 2007 that focused on the general characteristics, reproducibility, validity and feasibility of MS used in the assessment of PA (appraisals of three pedometers and nine accelerometers). Based on their analysis, they suggested MS to be sufficiently valid to assess PA in youth, and that most MS that they assessed were reliable enough. They also noted that using MS as an estimator of energy expenditure needed caution, due to the significant under- and overestimation when compared with the doubly-labeled water technique, a method that is considered a “gold standard” of energy expenditure estimation of free-living PA [10]. Although doubly-labeled water is accurate, its drawbacks are that it is expensive; it does not provide information about the intensity, frequency, duration, or type of PA; and it requires specific expertise [15]. Consequently, the last decade has witnessed an increase in the use of MS (pedometers, accelerometers) as PA assessment tools [14].

The pedometer is a simple MS device based on bodily acceleration or deceleration in one direction. It enables the observer to measure the number of steps taken over a period of time. The disadvantages of this technique are the inability to measure the intensity of PA; record the amount of PA (in terms of step counts) over several time periods; and document any energy expenditure increase achieved through carrying a load or walking/running uphill [3,16,17]. Nevertheless, researchers [14,17] have signified pedometers to be sufficiently valid and reliable to assess free-living PA in cases where the intensity of PA is not required.

The accelerometer is another type of MS used to assess PA; it is more sophisticated and provides more detailed outputs in comparison with pedometers. However, when compared with pedometers, the disadvantage of accelerometers is their price, which can be a limiting factor particularly for wide-scale investigations that recruit many participants. Hence pedometers have become an appropriate alternative when researchers seek to measure only the number of steps achieved [5].

As regards to MS, the Yamax Digi-walker is the most verified pedometer while the ActiGraph is the most verified accelerometer [14]. MS have the additional advantages of being light in weight and relatively low priced when compared with objective techniques (indirect calorimetry or doubly-labeled water) [18]; and that concurrently within their development they were validated and calibrated in various conditions.

As the internal mechanisms of such MS devices are different, the reliability and validity of each type of device is also different. Although such differences in qualities were of less significance in controlled conditions [13], more often such differences in qualities were important when free-living PA was being assessed [6,11,19–21]. Thus it is important to ascertain the extent of agreement (and disagreement) between pedometers and accelerometers in documenting the levels of free-living PA in different settings. In order to address this gap, the present study employed a sample of free-living adolescents in Poland and compared the Yamax Digi-walker (the most verified pedometer) and the ActiGraph (the most verified accelerometer).

### 1.1. Aims of the Study

This study assessed and compared, in a sample of free-living adolescents (N = 135), the daily step counts recorded by two different MS that operate with different principles and are both commonly employed in measuring the number of steps. We appraised the level of agreement of step counting of two MS: pedometer (Yamax Digi-walker SW-701) and accelerometer (criterion measure; ActiGraph GT1M). The specific objectives were to:

- assess the step counts recorded by each of the two MS for the same participant everyday over a period of one week;
- compare the step counts recorded by each of the two MS for the same participant in order to detect any inter-device differences on each day over a period of 1 week, employing the accelerometer as the criterion measure; and,
- classify the step counts recorded by each of the two MS for the same participant into PA categories, and compare these PA categories for the same participant in order to detect any inter-device differences in the categories on each day over a period of 1 week.

## 2. Methods

### 2.1. Participants, Settings and Procedures

As only 149 pairs of MS (pedometers and accelerometers) were available for the study, and we wanted to measure all participants in one ‘batch’ at one time, this limited the maximum sample size of participants that could be recruited to 149 pupils (mean age 18.0 ± 0.6 years; BMI range: 16.5−29.3 kg·m^−2^). Hence only four high schools were selected to implement the study, and the selection was based on successful previous collaborations between the Center of Kinanthropology Research at Palacky University (Czech Republic) and the schools. The schools had similar study curricula, and were all in urban areas in the Katowice region in Poland that had similar conditions for PE and free-living PA (Dabrowa, Katowice, Sosnowiec, and Swietochlowice; 64% of participants lived in cities populated by >100,000 inhabitants, 7% lived in cities populated by <30,000 inhabitants, and the rest of the sample lived in cities that were in between these two extremes). For instance, the four schools provided the same quantity of physical education (three hours of physical education per week), and had similar material facilities for PA (e.g., indoor and outdoor sport and exercise facilities *etc*.). None of the participants was obese or a professional athlete, and none of them had any mobility-impairing disabilities. About 8% of the participants were engaged in organized PA more than four times a week.

The Ethics committee at the participating Universities (in the Czech Republic and in Poland) agreed to the proposed research and approved the study. At the four sites, the aims and objectives of the study were clarified to all pupils who were then invited to participate in the research if they and their parents agreed (signed written informed consent). Participation was voluntary and pupils could withdraw from the study at any time. In order to be enrolled in the study, pupils had to agree to wear two MS devices continuously for the period of monitoring (one week). Equal numbers of participants were randomly selected from classes at each of the four schools. Data were anonymous and confidential, and data protection was observed at all times.

We only selected female pupils from the four schools in agreement with others that girls were more responsive than boys in wearing a pedometer [22]. Hence our sample comprised 149 girls who concurrently wore two MS: a Yamax Digi-Walker SW-701 (Yamax Co., Yasama Corp., Tokyo, Japan) pedometer (PDW), and an ActiGraph GT1M (Manufacturing Technology Inc., FL, USA) accelerometer (AAG) over a 7-day period in October 2008. The accelerometer was set according to the manufacturer’s specifications: it was calibrated and programmed to record information in 1-minute epochs (*i.e.*, the normal factory default sensitivity as set by the manufacturer). Participants wore both devices on opposite sides of the waist (located in pouches firmly fixed on elastic fabric belt) for a mean of 14.06 ± 2.13 hours per day (range = 8.98 to 19.77 hours per day), taking it off when sleeping and for hygiene (e.g., bathing, *etc*.). We excluded data from 10 girls due to technical causes (e.g., when any of the two devices had been left lying for some time or when a device was lost); and from another four girls due to record failures (data were considered incorrect if it was out of range of 1,000–30,000 steps per day). Thus, the present analysis employed data from 135 girls only.

### 2.2. Variables (Step Counts per Day; and Categories of PA)

When participants removed their PDW every evening, they recorded the data from the PDW monitor on a special data record sheet that we provided and then reset the pedometer to zero. Data from the AAG was retrieved by the special ActiPA2006 software [23]. Then, four sets of information were retrieved from the original data: (1) daily step counts achieved in each of the seven days of the monitoring week (Monday to Sunday); (2) daily step counts achieved within the whole week; (3) daily step counts achieved within the period of five work-days; and, (4) daily step counts achieved within the period of the weekend. When one of the paired values (readings from one MS) was missing for a given day for a given participant, the participant was excluded from the particular analysis. Then, for each of the two MS we employed, based on the recorded daily step counts, participants were grouped into five categories of PA, in agreement with published threshold guidelines [1]. The categories comprised: ‘sedentary’ (<5,000 steps/day); ‘low active’ (5,000−7,499 steps/day); ‘somewhat active’ (7,500−9,999 steps/day); ‘active’ (10,000−12,499 steps/day); and, ‘highly active’ (≥12,500 steps/day).

### 2.3. Statistical Analysis

Data was analyzed using STATISTICA 8 (StatSoft Inc., Tulsa, OK, USA) with significance level set at *p* < 0.05. As the data was not normally distributed for most variables, descriptive statistics are presented as medians (Mdn) and inter-quartile ranges (IQR). Correlation analyses (Spearman’s rank correlation coefficient) quantified the linear relationship between step counts recorded by the pedometer (steps per day_PDW_) and accelerometer (steps per day_AAG_) in all the individual days, in the 2-day period (weekend), in the 5-day period (workdays), and in overall 7-day period (week days). Spearman’s rank correlation coefficient was also used to assess the relationship between inter-device differences in step counts and the number of hours participants wore the devices. Kruskal-Wallis ANOVA tested whether inter-device differences were associated with particular schools (undertaken separately for all individual days, 2-day period, 5-day period, and 7-day period).

For the actual number of steps achieved, Wilcoxon-matched-pair test was used to verify non-zero differences between step counting of the two MS. Effect size [24] was then computed in order to assess the ‘practical’ significance of inter-device differences, employing the formula: $d=2\cdot \frac{Z}{\sqrt{N}}$, where *Z* was calculated from Wilcoxon-matched-paired test that was used to compute statistical significance. When we further classified the actual number of steps achieved into corresponding PA categories, intra-class correlation (ICC) appraised the degree of agreement of participants’ PA categorization that were derived from amount of steps obtained from PDW and AAG.

## 3. Results

Kruskal-Wallis ANOVA indicated that inter-device differences in steps counts were independent of the school where participants were recruited from (in all cases *p-levels* were at least ≥ 0.15). Likewise, the correlations between inter-device differences and duration of wearing the devices throughout the day were consistently low, and statistically non-significant in all the individual days, and in the 2-day, 5-day, and 7-day periods (*r_sp_* ranged between 0.07_5-day period_ to 0.14_2-day period_; in all, *p* ≥ 0.10).

Table 1 depicts that collectively in the total 7-day period of monitoring, depending on the MS, girls accumulated Mdn_AAG_ = 8,874 (IRQ = 6,695−10,777) steps per day, and Mdn_PDW_ = 8,489 (IRQ = 6,647−11,120) steps per day. For both devices, inter-day differences (steps per day) for individual days were significant in many instances (*p* < 0.05 in 9 out of the 15 possibilities that existed to compare the individual 7 days). Furthermore, for both MS, the differences between number of steps achieved in the periods ‘workdays’ and ‘weekend’ (inter-period differences) were also significant. Inter-device differences (steps per day) were significant only on Wednesday (*p* = 0.025) and Sunday (*p* = 0.036), and there were no inter-device differences for the periods ‘workdays’, ‘weekend’, and ‘whole week’. For the individual days where the differences between the two MS were significant, the effect size (*i.e.*, ‘size’ of the difference between the two devices) ranged from 0.36 (Sunday) to 0.39 (Wednesday) (*i.e.*, mostly poor to small effect) (Table 1). Furthermore, none of the differences (in steps per day) between both devices exceeded 1,000 steps per day—a level that might be insufficient for some authors to consider such differences to be of ‘practical’ significance (we based ‘practical’ significance on the rationale that others [1] employed levels of 2,500 steps per day as differences between successive incremental categories of PA levels).

Table 2 shows that across each of the seven individual days, in the 5-day period (workdays), in the 2-day period (weekend), and in the overall 7-days period (whole week), the correlations between the two MS were positive, moderately or slightly high correlated (*r_sp_* range = 0.65 to 0.74), and significant (*p* < 0.001).

We then classified the step counts recorded by each of the two MS for the same participant into categories, and compared these categories for the same participant in order to detect any inter-device differences in the categories.

Using the pedometer-determined step counts to generate the PA categories, 7.4% of our girls were classified as ‘sedentary’; 25.9% as ‘low active’; 33.3% as ‘somewhat active’; 22.2% as ‘active’; and 11.1% as ‘highly active’ in the whole 7-day period of monitoring. Table 3 illustrates that across each of the seven individual days, the ICC for the participants’ PA categorization based on PDW- and AAG-counted steps, varied from 0.69 to 0.62; and within the 2-day, 5-day, and 7-day periods, the ICC ranged from 0.64 to 0.63.

Table 4 depicts the levels of agreement and disagreement between the PA categories (based on step counts) generated from the data recorded by the two MS (pedometer and accelerometer). Each PA category represented 2,500 steps.

*Agreement of PA categories generated by each MS*: section (A) of Table 4 shows that there was fair total agreement between the two devices (range: 43−60% total agreement) in classifying each participant’s step counts into PA categories as recorded by each MS for the same participant.

*Disagreement of PA categories generated by each MS*: when the PA categories generated from the step counts recorded by the two MS were different (*i.e.*, disagreed), we further assessed the direction and size of the disagreement *i.e.*, whether the pedometer generated an underestimation or overestimation in comparison to the accelerometer. Section (B) of Table 4 shows these disagreements (under- and overestimations) grouped by the number of categories difference between the two MS. For both the under- and overestimations, the most frequent disagreements were those where the differences comprised one PA category, followed by disagreements where the differences comprised two categories. It was reassuring to find that disagreements of three PA categories were relatively less common, comprising 1−4% of all cases; and disagreements of four categories were very few (range: 1−2% of all cases). No distinct pattern was observed in the total % of participants underestimated or overestimated across a particular time period or by workday or weekend. However the greatest disagreement (combined under- and overestimations) for the time periods was for the 5-day monitoring (57%); for the individual days, the greatest disagreement was on Monday (52%) followed by Wednesday (50%).

*Disagreement of more than one category of PA*: when the disagreement between the pedometer and accelerometer was of >1 PA category, we undertook no further analysis as such disagreements were deemed important because they have practical implications.

*Disagreement of only one category of PA*: when the disagreement between the two MS was only by 1 PA category, in order to quantify the size of any given extent of disagreement (regardless of whether it was an underestimation or overestimation), we further sub-divided only those disagreements of one PA category into four sub-groups based on the size of the differences in the number of steps recorded by each of the two devices. As each PA category comprised 2,500 steps, four ‘disagreement groups’ were constructed (Section C of Table 4): disagreement up to ± 1% of the size of the category (*i.e.*, difference of up to ± 25 steps); disagreement up to ± 5% (*i.e.*, difference of up to ± 125 steps); disagreement up to ± 10% (*i.e.*, difference of up to ± 250 steps); and, disagreement > ± 10% (difference > ± 250 steps).

This procedure enabled us to ‘gauge’ when a given 1-category disagreement was due to two recorded step counts (pedometer and accelerometer) that were close to but on the opposite sides of the border of the given PA category *i.e.*, when the differences were relatively small—‘borderline cases’ e.g., ± 1% (difference of ± 25 steps) or ± 5% (difference of ± 125 steps); or alternatively, when the differences were larger—‘non-borderline cases’ e.g., up to ± 10% (difference of ± 250 steps), and disagreement > ± 10 % (difference > ± 250 steps). Such distinction is important as the existence of many of such ‘borderline cases’ could lead to misinterpretation (a further overestimation) of the total disagreement values.

Section (C) of Table 4 details the analysis of all 1-category disagreements *i.e.*, differences that could have been due to the step values of each of the two MS falling close to either side of the border of a given PA category. The findings suggested only a few ‘borderline cases’: none of the sample had differences that were up to ± 1% (*i.e.*, up to ± 25 steps), and only 1% of the sample showed differences comprising up to ± 5% disagreement category size (*i.e.*, up to ± 125 steps). Section C also depicts that about 3% of the sample displayed ‘non-borderline cases’ (those comprising up to ± 10% disagreement *i.e.*, up to ± 250 steps). The highest frequency of disagreements were those where there were > ± 10% disagreement (> ± 250 steps respectively). For instance 47% of all participants were classified differently by the two devices in the 7-days period, and for all these 47% of participants, the disagreement was > ± 250 steps (last row of the Table 4). Similarly 57% of all participants were classified differently by the two devices in the 5-days period, and for all these 57% of participants the disagreement was > ± 250 steps. Across the individual days of monitoring, the disagreements of > ± 250 steps ranged from 39% (Thursday) to 48% (Monday). This suggested that most of the disagreements (in PA categories generated from the step counts recorded by each of the two MS) were due to ‘non-borderline cases’.

**Figure 1.**
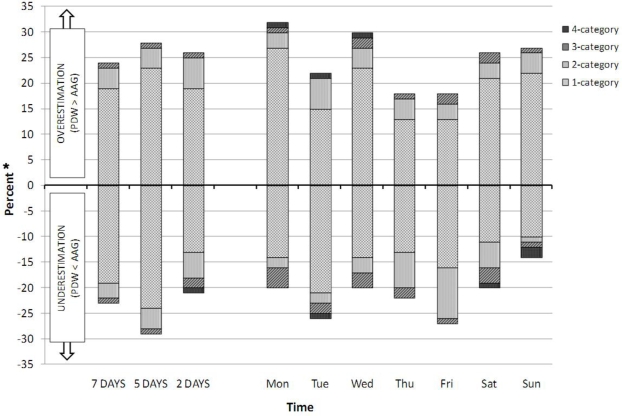
Direction and amount of disagreement of participants’ PA categorization based on pedometer (PDW) when compared to accelerometer (AAG) step counts (N = 135). Note: 1-category: inter-device difference within the range of one PA category (± 2,500 steps); 2-category: inter-device difference within the range of two categories (± 5,000 steps); 3-category: inter-device difference within the range of three PA categories (± 7,500 steps); 4-category: inter-device difference within the range four PA categories (± 10,000 steps);* percent of participants that were over or under estimated by PDW.

Figure 1 illustrates the extents and directions (over- and underestimations) of disagreement between the pedometer and accelerometer when the numbers of steps recorded by each were translated into participants’ PA categories. The 7-day period (first column in Figure 1) depicts relatively equal ratios of over- and underestimation of the pedometer as regards the percentages of total disagreement (*i.e.*, all the categories of disagreement combined). The 7-day period also depicts relatively equal ratios of over- and underestimation of the pedometer as regards the percentages of disagreement in the *individual* PA categories. These near-equal ratios of over- and underestimations of the pedometer when compared with the accelerometer give the first column of Figure 1 a ‘mirror image’ appearance on both sides of the x-axis (the column’s height and composition are nearly the same in both directions). However, the 7-day period represented a ‘summary’ of all the days of the week, and a detailed view of the individual days revealed some discrepancies when compared with the 7-day period. Indeed across the individual days, we noted three points: the unequal lengths of the bars on both sides of the x-axis (unequal *total* disagreement regardless of whether it is was under- or overestimation); there was not a very clear ‘mirror image’ of near-matching extents of over- and underestimations (unequal lengths of the overestimations and underestimations bars for any individual day); and within the over- or underestimations, there was unequal ratio of the difference categories that comprised the total over- or underestimations (unequal lengths of the subparts that comprise the total overestimation or underestimation bars for any individual day).

## 4. Discussion

There is increasing interest in objective monitoring of daily PA using electronic MS, e.g., accelerometers and pedometers [25–28]. However the validation of any MS should be undertaken in field settings employing direct observation or doubly-labeled water as criterion measures [29]. Nevertheless, for large studies, these ‘gold standard’ criteria are expensive and require time. Hence the present study concurrently compared an accelerometer and a pedometer as regards two measurements undertaken by each MS (step counts recorded; and categories of PA calculated). This research contributes to further expand our understanding of the differences in measurements recorded by pedometers and accelerometers (the latter used in this study as the criterion measure). Others have reported the Yamax (SW701) pedometer as an acceptable criterion pedometer [11]. We build on such findings and assessed this Yamax pedometer *vis-a-vis* an accelerometer as a criterion.

As regards the first objective of the study, we appraised the step counts recorded by each of the two MS for the same participant everyday for a week. The step counts of 135 adolescent girls as measured by two MS showed relatively high variability of their PA (IQR range = 4,053 to 6,698 steps per day, Table 1) across the monitored days and the time periods. Mean number of steps recorded by our participants across the week ranged between 6,753–9,414_pedometer_ and 6,410–10,043_accelerometer_. When these *step counts* were compared to the guidelines of the 2001–02 President’s Challenge Physical Activity and Fitness Awards Program (11,000 steps per day for girls [30]), our sample appeared not to meet this recommended guideline. However, a point to note is that the recommendations of the President’s Challenge Physical Activity and Fitness Awards Program [30] were derived from an American population sample. Nevertheless, in the monitored week, regardless the type of MS (pedometer or accelerometer) that recorded the number of steps, the PA levels of these adolescent girls were mostly ‘somewhat active’ as classified by a highly cited study [1], or as used by Hatano [31]. Interestingly, the PA levels of our adolescent girls were classified as ‘good’ PA level as used by Sigmund and co-researchers [32]. However they [32] used a scale where ‘good’ represented the 4^th^ highest level out of six levels.

Regardless of the type of MS, we found significant inter-day differences (across the week) and also significant work- and weekend days differences in the recorded number of steps. Such differences highlight the peculiarity of individual days in relation to the amount of PA achieved by people. These day-to-day differences in PA (more active in weekdays than weekend; least active on Sunday in comparison to the rest of days of the week) are in agreement with others [22,33–38]. People view the weekend as a relaxation time and hence may not push themselves as hard on the weekend [39]. These findings further confirmed that the assessment of PA levels could be strongly dependent on the given day on which PA monitoring is performed. Such features would need to be considered in the research design of studies where free-living PA is evaluated. There seems a lack of consensus of opinions about the day of the week where the highest step counts are usually attained [39,40–43].

As regards the step counts achieved, in relation to the study’s second objective, we compared the number of step recorded by each of the two MS for the same participant in order to detect any inter-device differences on each day over one week. As presented in the results section, the correlations between inter-device differences and the duration of wearing the devices throughout the day were low, suggesting that our findings of the inter-device differences were not influenced by the duration of wearing the devices. Only on two days of the week (Wednesday and Sunday), the numbers of steps recorded by each MS were significantly different (inter-device differences, where the pedometer overestimated the number of steps in comparison to the accelerometer) (Table 1). However, for the whole 7-day period (and for workdays and weekend) there were no significant differences in the number of steps recorded by the two MS, denoting sufficient accordance of both devices. In this sense, we could recommend the use MS (for monitoring the step counts achieved by free living people) when the duration of monitoring is for some longer time period (week, workdays or weekends) rather than on single individual days only.

The mostly positive differences of the measured step counts (differences of devices’ medians: Mdn_AAG_–Mdn_PDW_, Table 1) indicated that the pedometer (PDW) underestimated the number of steps in comparison with the accelerometer (AAG) as a criterion measure. This is in accordance with Le Masurier *et al*. [20] who reported the underestimation of the Yamax SW200 pedometer (in comparison with AAG) under controlled conditions, and the decrease of such underestimation with the increase of the speed of locomotion. We are also in agreement with others [13,44] who have reported similar results: that pedometer underestimated the number of steps achieved when compared with accelerometer.

In our sample, the mostly small effect size (size of the differences, regardless of whether it was under- or overestimation) seems to provide some evidence for the agreement of both devices as step counters. However, we found that the inter-device correlations were not so high (Table 2). Indeed our correlation between the two devices in the 7-day period (*r_sp_* = 0.65) was mostly lower when compared with other studies that employed exactly the *same* brands of MS (as those that we used) to measure convergent validity of these MS in different populations. For instance, our value (0.65) was lower than in Belgium where *R* was 0.73 for 76 children aged 4–5.9 years (monitored for 5days) [16], and also lower than in Great Britain where *R* was 0.86 for 121 older persons > 65 years (monitored for 7 days) [10]. However our correlation findings between the 2 devices were close to that reported in Texas, USA where *R* was 0.60 for 78 children 11−15 years. However the Texas study was under controlled conditions rather than for free-living PA [45].

Further, our correlation between the two devices in the 7-day period (*r_sp_* = 0.65) was also mostly lower when compared with other studies that assessed *different* models of Yamax pedometer (e.g., Yamax SW200, SW500) that the one we used (Yamax SW701) in relation to different brands of accelerometer (other than the Actigraph GT1M which we used). For instance, authors have reported higher convergent validity of Yamax pedometers (SW200, SW500) with various types of accelerometers in adults, e.g., with the Tritrac-Vmag accelerometer, *R* = 0.93 [46] or CSA accelerometer, *R* = 0.86 [21, 46]. An explanation for the partial divergence of our findings (our lower correlations between both MS that we used) in relation to other studies could be a consequence of the specific characteristics of PA of the observed population and the fact that we observed a relatively homogeneous sample where there has been reports to suggest that the correlations in homogenous samples could be lower than in non-homogenous samples [47]. Reliability and (more so) validity of frequently used MS depend on the type of PA [21,48] and its intensity [9]. Therefore the preferred type of PA, the levels of various habitual activities (including occupation), and their different intensities (e.g., variance due to gender [49,50] or age [50]) in an observed population have strong relationships to the level of validity of a MS device. Although the correlation between the two MS in our study was relatively lower than others e.g., [10,16], due to the range of afore-mentioned reasons, the MS we assessed can be considered as useful devices to measure the amount of steps of adolescents’ free-living PA.

As regards the PA category achieved, in connection with the study’s third objective, we classified the step counts recorded by each of the two MS for the same participant into five PA categories, and compared these categories for the same participant in order to detect any inter-device differences in the categories on each day over a period of one week. Although PA categorization based on step counting has been used when assessing the PA levels of free living people [51–54], our ICC values (and their 95% confidence intervals) of the PA categories computed from the step counts of both MS indicated low agreement between the PA categories generated by each of the two devices (ICC = 0.62−0.67). However, ICC values are influenced by many factors (e.g., age; gender; sample size; number of devices that are being compared, *etc.*) [55]. The low ICC between the two devices in our study (0.62−0.67) is further supported by our finding of low (43% to 60%) inter-device agreement of the two MS in categorizing our participants’ PA (into ‘sedentary’, ‘low active’, ‘somewhat active’, ‘active’, and ‘highly active’ categories). It is difficult for us compare our PA categories findings with others as most studies [14,17,45,56,57] compared the actual number of steps and did not go further to actually compare the PA categories that are generated from these reported number of steps.

When the PA categories generated from the data recorded by the 2 MS were different (*i.e.*, disagreement), we assessed whether the pedometer generated an over- or underestimation in comparison to the accelerometer, and the size of the disagreement. Figure 1 suggested that for the individual days, it was not easy to indicate precisely whether the pedometer generally over- or underestimated the PA categories in comparison to accelerometer. However, for the overall 7-day period, there were comparatively ‘balanced’ extents of over- and underestimation of the pedometer in respect to the accelerometer (more symmetrical ‘mirror image’ appearance of the column on both sides of the x-axis). Conversely, the more ‘asymmetric’ columns (in terms of unequal over- and underestimation) were Monday, Wednesday and Sunday. Moreover, Monday and Wednesday were the days with the highest total disagreement; Wednesday and Sunday were the only days with statistically significant inter-device differences in number of steps; and in all three days (Monday, Wednesday and Sunday), the pedometer had the most tendencies to overestimate the PA categories. These findings might suggest some special circumstances/ characteristics of these days (e.g., weather conditions), or alternatively that these differences could be random findings. Further research would confirm or refute such findings.

Our most frequent inter-device disagreements were those where the differences comprised one PA category, followed by disagreements where the differences comprised two categories, with very few cases of disagreements of three and of four categories. Disagreements of *more than* one PA category clearly have practical implications when monitoring adolescents’ PA. Similarly, disagreements of *only* one category could have practical implications if the values generated by the two MS do not lie close to either side of a given category’s boundary *i.e.*, not borderline. We found very few such ‘borderline cases’: none of the sample had small differences (up to ± 25 steps), and only 1% had differences comprising up to ± 125 steps. About 3% of our adolescents displayed ‘non-borderline cases’ (those comprising up to ± 250 steps), and the highest frequency of disagreements were those that comprised > ± 250 steps. For instance 47% of all participants were classified differently by the two MS in the 7-day period with the magnitude of disagreement being > ± 250 steps.

Collectively, these results suggested that the disagreement between the two devices in categorizing PA was not mainly due to some values lying close to but on opposite sides of the borders of a given PA category. Rather, most of the disagreement was due to values lying away from and on opposite sides of the borders of a given PA category (non-borderline cases). When this finding is coupled to the fact that we found many high disagreement instances (when the inter-device disagreement was >1 category), this suggested that caution need to be exercised when ‘translating’ pedometer or accelerometer step counts into corresponding PA categories. It was not feasible for us to compare our PA categorization findings as most research only compared step counts rather than PA categories [10,14,16,18,19,57].

Hence our findings suggested that the likelihood of ‘erroneous’ PA categorization (disagreement of categories generated by each MS) for the same participant was not low. Nevertheless, obtaining and assessing information about a person’s PA category is valuable and in many instances such PA category information is combined with other outputs of complex assessments. This proposed that caution needs to be exercised in the interpretation of such PA category data. Our findings also suggested that it could be advantageous to wear two MS devices concurrently in PA research. Concurrent usage of monitors provides more complex and more specific information about the ‘real’ amount of performed PA. It also allows a person’s PA categorization to be made on the basis of steps number obtained from PDW and AAG. Further, wearing both devices facilitates immediate feedback (pedometer) [58–60], and also consequential education-motivated feedback (accelerometer) [61] to the participant. However, the actual price of an accelerometer is several-fold more than the price of a pedometer, a point that may evocate rejection of the concurrent wearing of two MS.

This study has limitations. The data is from girls from four schools in one country. Participants were monitored during autumn, and the structure and amount of PA could be different than during others seasons [39]. Some movement assessment devices have the draw back that their measurements could be impeded when the position of the accelerometer, which should be vertically aligned, is changed in position by soft tissue in the waist region (the place of attachment of the device). Measurements can be affected by such repositioning and might have contributed to the differing results. Further, pedometers are incapable of measuring activity intensity and underestimate the number of steps taken at slower walking speeds, so measurements/ indications of the walking speeds would have been useful.

Although the pedometer is rapidly becoming the preferred objective technique of measuring PA in large populations, previous studies have focused on the quality (e.g., differences in the validity or reliability) of the different brands of pedometers, and have reported non-uniform findings [14]. We found mostly non-significant differences in step counts between the pedometer and accelerometer. Future research could provide more detailed analyses of the factors that might have a negative influence on the validity of pedometer step counting. Further, the immediate feedback that participants received from pedometers could lead to an increased motivation for PA [60], therefore a pedometer’s motivational effect size should be described and analyzed.

## 5. Conclusions

The present study advances our knowledge about MS (pedometer and accelerometer) used for objective assessment of PA. Our sample of Polish adolescent girls PA levels fell mostly in the middle of the PA classifying scale (as “somewhat active” or as “good”), but the variability of recorded PA (steps counts) was relatively high. The girls’ PA was not of the same level in all the days of the week; the day-to-day differences and also the differences between weekdays and weekend were statistically significant.

The inter-device comparison of steps achieved showed significant differences on Wednesday and Sunday. There was moderate inter-device correlation for the number of steps achieved on all the days, as well as in the time periods (week, weekdays, and weekend). Using the accelerometer as the criterion, the concurrent validity of the Yamax SW-701 pedometer was considered to be low. However caution needs to be taken when interpreting the findings, as the validity of MS varies with different populations, type of preferred PA, intensity of PA, accessibility of PA and others factors. When these are taken into account, we consider the Yamax SD-701 to generate valuable step count information and to be a useful device to measure the amount of steps achieved by free-living adolescent females.

The total values of the differences in number of steps mostly indicate that the Yamax SW-701 pedometer generally underestimated the step counts in comparison with the ActiGraph GT1M accelerometer, although when there were significant differences between the two MS, the pedometer usually overestimated the step counts.

The likelihood of ‘erroneous’ PA categorization (disagreement of categories generated by each MS) was high. This fact raises two questions: the translation of pedometer and accelerometer step counts into corresponding PA categories; and the ‘credibility’ of the use of five-category classification of PA (sedentary, low active, somewhat active, active, and highly active). Caution is recommended when using such PA categories in the assessment of people’s free-living PA.

MS should be used as PA assessment tools more safely in longer-term periods of monitoring (at least seven days); as in the case of individual-day monitoring, there is the likelihood of significant inter-devices differences that could lead to erroneous interpretation of results. Hence the concurrent usage of both MS could provide more complex information about adolescents’ PA, and could assist in immediate feedback to participants (by pedometer) followed by education-motivate feedback (by accelerometer).

## Figures and Tables

**Table 1.** Median number of steps achieved: Inter-device differences between pedometer and accelerometer (N = 135).

**Period of measurement**	**Pedometer PDW**	**Accelerometer AAG**	**Inter-device difference**		

	**Mdn (IQR)**	**Mdn (IQR)**	**Diff_(median)_**	**d**	**p-value**
**Individual days**					

Monday	8,593 (6,155; 11,546)	8,915 (6,289; 11,455)	322	0.14	0.131
Tuesday	8,372 (6,016; 11,581)	8,565 (5,809; 10,782)	193	0.07	0.684
Wednesday	9,414 (7,212; 11,884)	9,051 (6,876; 11,689)	−363	0.39	0.025*
Thursday	7,176 (4,978; 10,204)	7,527 (5,691; 10,064)	351	0.13	0.434
Friday	9,074 (6,883; 13,581)	10,043 (7,296; 12,622)	969	0.07	0.702
Saturday	8,049 (5,185; 11,804)	8,929 (5,844; 12,186)	880	0.02	0.926
Sunday	6,753 (4,247; 10,610)	6,410 (4,082; 9,722)	−343	0.36	0.036*

**Time periods**					

5-day period (Workdays)	9,094 (6,910; 10,963)	9,098 (6,927; 11,132)	4	0.15	0.397
2-day period (Weekend)	7,667 (5,372; 10,620)	7,772 (5,607; 10,745)	105	0.18	0.300
7-day period (Whole week)	8,489 (6,647; 11,120)	8,874 (6,695; 10,777)	385	0.14	0.423

PDW: pedometer (Yamax Digi-walker SW-701); AAG: accelerometer (ActiGraph GT1M); Mdn: median; IQR: inter-quartile range; Diff_(median)_ = Mdn_AAG_ - Mdn_PDW_: inter-device difference of medians;

* statistically significant inter-device difference (Wilcoxon-matched-pair test); d: effect size.

**Table 2.** Number of steps achieved: Inter-device correlation between pedometer and accelerometer.

**Number of steps achieved on individual days**	**r_sp_**	**p-value**
Monday	0.68	< 0.001
Tuesday	0.66	< 0.001
Wednesday	0.65	< 0.001
Thursday	0.69	< 0.001
Friday	0.72	< 0.001
Saturday	0.69	< 0.001
Sunday	0.74	< 0.001

**Time periods**		

5-day period (workdays)	0.64	< 0.001
2-day period (weekend)	0.66	< 0.001
7-day period (whole week)	0.65	< 0.001

N = 135; r_sp_ – Spearman’s rank order correlation coefficient.

**Table 3.** Category of PA achieved: intraclass correlation between participants’ PA categorizes based on pedometer and accelerometer step counting (N = 135).

**Category of PA achieved on**	**ICC**	**95% CI**
**Individual days**		

Monday	0.65	0.54; 0.73
Tuesday	0.63	0.52; 0.72
Wednesday	0.62	0.50; 0.71
Thursday	0.69	0.59; 0.77
Friday	0.69	0.59; 0.77
Saturday	0.68	0.58; 0.76
Sunday	0.69	0.59; 0.77

**Time periods**		

5-day period (workdays)	0.63	0.51; 0.72
2-day period (weekend)	0.62	0.50; 0.71
7-day period (whole week)	0.64	0.53; 0.73

ICC: intraclass correlation; CI: confidence interval.

**Table 4.** Agreement and disagreement between participants’ PA categorization based on pedometer (PDW) and accelerometer (AAG) step counts (N = 135).

Differences in Step Count Categories*	**Duration of Monitoring**									

	**Time Periods**			**Individual Days**						

				**Workdays**					**Weekends**	

	**7 days**	**5 days**	**2 days**	**Mon**	**Tue**	**Wed**	**Thu**	**Fri**	**Sat**	**Sun**
**AGREEMENT (PDW=AAG)**										

**A. TOTAL AGREEMENT**	**53**	**43**	**52**	**48**	**52**	**50**	**60**	**54**	**53**	**57**

**DISAGREEMENT (PDW ≠ AAG)**										

**B. TOTAL DISAGREEMENT**	**47**	**57**	**48**	**52**	**48**	**50**	**40**	**46**	**47**	**43**

**Underestimation (PDW <AAG)**										

**Total % of participants underestimated by PDW†**	23	30	21	20	26	20	21	28	21	15

1 category difference	19	24	13	14	21	14	13	16	11	10
2 category difference	3	4	5	2	2	3	7	10	5	1
3 category difference	1	1	2	4	2	3	2	1	3	1
4 category difference	—	—	1	—	1	—	—	—	1	2

**Overestimation(PDW> AAG)**										

**Total % of participants overestimatedby PDW‡**	24	27	27	32	22	30	19	18	26	28

1 category difference	19	23	19	27	15	23	13	13	21	22
2 category difference	4	4	6	3	6	4	4	3	3	4
3 category difference	1	1	1	1	—	2	1	2	2	1
4 category difference	—	—	—	1	1	1	—	—	—	—

**C. Combined Underand Overestimation (PDW≠ AAG by 1 category)**										

**TOTAL DISAGREEMENT**	**47**	**57**	**48**	**52**	**48**	**50**	**40**	**46**	**47**	**43**

Disagreement up to ± 1% (difference of up to ± 25 steps)	—	—	—	—	—	—	—	—	—	—
Disagreement up to ± 5% (difference of up to ± 125 steps)	—	—	—	1	—	—	—	—	—	—
Disagreement up to ± 10% (difference of up to ± 250 steps)	—	—	—	1	—	—	1	—	1	—
Disagreement > ± 10% (difference > ± 250 steps)	47	57	48	51	48	50	39	46	46	43

All cells are percentages of participants;

* each category represents 2,500 steps (sedentary: < 5,000 steps/day; low active: 5,000–7,499 steps/day; somewhat active: 7,500–9,999 steps/day; active: 10,000–12,499 steps/day; and highly active: ≥ 12,500 steps/day);

† total percentages of participants where PDW < AAG by at least one category;

‡ total percentages of participants where PDW > AAG by at least one category.
